# Newborns' clinical conditions are correlated with the neonatal assessment manual scorE (NAME)

**DOI:** 10.3389/fped.2022.967301

**Published:** 2022-09-09

**Authors:** Andrea Manzotti, Francesco Cerritelli, Erica Lombardi, Simona La Rocca, Pamela Biasi, Marco Chiera, Matteo Galli, Gianluca Lista

**Affiliations:** ^1^Research and Assistance for Infants to Support Experience (RAISE) Lab, Foundation Centre for Osteopathic Medicine (COME) Collaboration, Pescara, Italy; ^2^Division of Neonatology, “V. Buzzi” Children's Hospital ASST-FBF-Sacco, Milan, Italy; ^3^Research Department, SOMA Istituto Osteopatia Milano, Milan, Italy

**Keywords:** name, newborns, neonatal intensive care unit, pathologies, clinical conditions, prematurity, touch

## Abstract

**Objective:**

To investigate the relationship between the Neonatal Assessment Manual scorE (NAME) and newborns' clinical condition on a large number of infants. The NAME model was developed as an instrument to assess the infant's general conditions, especially in NICUs, by evaluating how the infant's body responds to an external stressor such as static touch. Previous studies, employing experienced assessors, showed good validity indices as well as high inter-rater reliability.

**Study design:**

Newborns were recruited at the “Vittore Buzzi” Pediatric Hospital NICU ward in Milan and their clinical conditions were collected through a standardized form—the complexity index. Two manual practitioners assessed all eligible newborns using the NAME scores. Data was analyzed using Kendall's τ correlation and odds ratio (OR) to assess the relationship between the NAME scores and the complexity index.

**Results:**

Two hundred two newborns (46% female; 34.1 w ± 4.3; birth weight of 2,093.4 gr ± 879.8) entered the study. The Kendall's correlation between the clinical conditions (complexity index) and the NAME score was −0.206 [95% CI: (−0.292, −0.116), *p*-value < 0.001], corresponding to an OR of 0.838 [95% CI: (0.757, 0.924), *p*-value < 0.001]. Further exploratory analyses showed significant correlation between gestational age, birth weight and NAME scores.

**Conclusion:**

The present paper adds evidence to the NAME model validity by demonstrating its applicability in the clinical neonatological context.

## Key points

- Complex newborns showed worse clinical conditions
- Complex newborns showed lower NAME scores
- The NAME could help recognize complex newborns
- Newborns' characteristics affected NAME scores

## Introduction

Infants' comorbidities in the neonatal intensive care unit (NICU) increase the complexity of caregiving. Maternal stress (1), labor and delivery complications (2, 3), and preterm births (4) are conditions that involve several pathologies that can threaten the babies survival (4). Indeed, clinicians need to pay particular attention to carefully monitoring infants' psychophysical conditions and creating adequate therapeutic plans to take care of them (5, 6). As a result, in the last decades, the advance in technologies and procedures improved infants' clinical conditions in NICU, showing a significant reduction of length of stay and mortality, specifically in premature babies (7).

Prematurity has a global incidence of about 11% (8) and is often accompanied with several comorbidities including respiratory distress, necrotizing enterocolitis, cardiovascular diseases, neurodevelopmental delay, reduced growth, sepsis, hearing, and visual impairments (4). In this complicated clinical status, babies who suffer from one or multiple pathologies, or who experience complications, require to be continuously evaluated to prevent adverse outcomes (8).

Recent studies investigated all the factors that could enhance the management of such critical conditions in premature babies, considering touch-related procedures with growing attention (9, 10). In fact, premature babies are touched and handled about a 100 times a day by doctors and nurses while performing routine-care procedures, including feeding, weighting, applying tubes, changing diapers, performing heel sticks, venipunctures, palliative care procedures, and managing emergencies (9, 11).

The role of touch in NICU has been investigated by several studies, which show the efficacy of different approaches–including massage therapy, kangaroo care, and osteopathic manipulative treatment–in positively affecting the regulation of oxygen saturation and heart rate in newborns and the survival and growth in preterm babies (12–22). Concerning assessment procedures, two scales, i.e., the Brazelton Neonatal Behavioral Assessment Scale and Assessment of Preterm Infants' Behavior, involve manual evaluation, but the palpatory findings are viewed as marginal aspects in a wider behavioral evaluation (23–25).

With the aim of proposing a rigorous and structured touch-based evaluation for newborns, a new model–the Neonatal Assessment Manual scorE (NAME)–was recently developed. It was conceived to be easily used by physiotherapists or manual therapists with experience in the pediatric field; furthermore, it was conceived to give a score that NICU professionals could easily use to understand and communicate about an infant's clinical conditions. The purpose of the NAME is to evaluate how newborns adapt and respond to a touch-based stimulus through the activation of their autonomic nervous system (ANS) and central interoceptive network. As a result, the NAME produces two scores: (1) one categorical, with three possible levels (“Bad,” “Marginal,” and “Good”), and (2) one numerical (a 1-to-9 Likert scale). Higher scores mean a better adaptive capacity of the newborn (10). In preliminary studies, this manual assessment procedure has been shown to be reliable, valid, easy to perform and safe. To date there are no studies which have investigated the relationship between the NAME scores and newborns' clinical condition on a large number of infants.

Therefore, the present study aimed to evaluate, on a larger sample, the relationship between the NAME and newborns' clinical conditions as routinely evaluated by nurses and/or neonatologists.

Should the NAME show an important correlation, then the NAME could become an important and useful clinical tool for all NICU professionals who are interested in obtaining information about the general conditions and adaptive status of newborns through an easy, valid and reliable manual approach.

## Materials and methods

### NAME description

The NAME model has been developed in the NICU ward, where newborns were assessed either in the incubators or beds. The NAME procedure consists in applying gentle tactile stimulations on the newborns: one hand is placed on the cranial region with the whole palm and the other hand on the sacral crest with the base phalanges (10). The operator—hat is, manual therapists experienced in the pediatric field—may change the position of the hands to adapt to the newborns' fragile conditions and the presence of neonatal support devices (10).

The operator then applies specific manual stimuli and focuses their attention on the sensory signals detected by their haptic system (e.g., touch and proprioception) (10). By evaluating the changes in the newborn's tissues, the operator aims to assess the newborn's compliance and homogeneity, i.e., briefly, how the newborn's body mechanically responds to gentle touch (10).

In fact, the kind of light touch used during the NAME procedure can stimulate both the Merkel-neurite complexes and the C-tactile fibers, which, as a consequence, release signal molecules that can influence blood circulation, pulmonary hemodynamics, smooth musculature, and the ANS (10). Therefore, the applied touch can induce changes in the cardiovascular and respiratory systems: as a consequence, *via* ANS, the central interoceptive network can redistribute the bodily fluids throughout the body modifying the local and global body volume. This modification changes the tissues texture, in particular, the softness or hardness of the tissues, which can be felt through haptic perception by an operator. Indeed, by positioning the hands around the baby's body—one hand around the cranial region and the other one around the sacral crest—the operator evaluates whether the bodily tissues are compliant with the mechanical stimuli given by the hands, that is, whether they change their texture according to the stimuli or if they put up some resistance. Since these responses are due to the ANS and the central interoceptive network functionality, which is affected by the infants development, growth and clinical status, assessing how the bodily tissues respond could give relevant hints regarding the infants' clinical conditions [see (10) for in-depth description of the NAME rationale].

A single application of the NAME evaluation lasts about 90 s and consists of two phases (10, 26). The first phase assesses the newborn's general compliance and lasts about 10 s. Using both hands, the operator applies a light pressure and then releases it in order to perceive the resistance that the newborn's body, as a whole, puts up against the manual pressure. The second phase assesses whether the newborn displays the same response throughout their body, that is, the newborn's body homogeneity (10). This phase lasts about 80 s: the operator applies the same light pressure as the first phase, but pays attention to whether there are body areas that react differently compared to others, since these could correlate with the newborn's clinical conditions (10, 26).

After the NAME evaluation, the operator assigns a categorical score (the main score with clinical usefulness as it aims to communicate about infants' conditions) which is then converted to a numerical one. The categorical score is one of three possible labels, i.e., “Bad,” “Marginal,” and “Good,” whereas the numerical one consists of a Likert scale ranging from 1 (worst possible score) to 9 (best possible score). In particular, the numerical score ranges from 1 to 3 for “Bad,” from 4 to 6 for “Marginal,” and from 7 to 9 for “Good” (Table 1).

**Table 1:** NAME scoring system.

**Categorical score**	**Numerical score**
Bad	1
	2
	3
Marginal	4
	5
	6
Good	7
	8
	9

Since the NAME procedure relies primarily on the haptic system of the operator, which can be affected by top-down processes and biases as every other sensory systems, it is paramount for the operator to be highly trained in touch-based procedures and to have years of experience in the neonatology ward to gain the necessary knowledge to better interpret the bodily tissues changes.

### Design of the study

The experienced manual professionals were identified among a cohort of physiotherapists and osteopaths having more than 10,000 h of clinical practice and specific training in the pediatric field. Two osteopaths with specific training in the pediatric field and more than 10 years of experience in the treatment of newborns were recruited and included in the study [age: 42 ± 7, Male (%): 1 (50), years of experience: 11.5 ± 7.68].

### Subjects

All newborns entering the “Vittore Buzzi” Pediatric Hospital NICU ward in Milan, Italy were considered eligible. The study period was from September 2018 to November 2019. The study was approved by the Hospital Ethics Committee (563-04/05/2018) and was conducted according to the Helsinki declaration.

Newborns were included if they fulfilled the following inclusion criteria: born between 29^+0^ and 36^+6^ weeks of gestation, either sex, birth weight >500 grams, stable clinical conditions (e.g., without respiratory or cardiovascular instability).

Newborns were excluded in case of gestational age <29 weeks or if critical clinical conditions were observed by the neonatologist in charge of population selection. Newborns for whom we lacked consent to the present study were also excluded.

For the whole duration of the present study, the enrolled infants continued the standard medical treatment. The NAME procedure was applied when the infants were asleep or in quiet wakefulness. Every enrolled infant was evaluated with the NAME procedure once and by one of the two recruited osteopaths.

### Outcomes

The primary outcome was the relationship between the newborns' clinical conditions as routinely assessed by the neonatologist and/or nursery and the NAME categorical score.

Secondary outcomes assessed through exploratory analyses were the correlation between, on the one hand, the demographic characteristics and clinical conditions of the infants and, on the other hand, the NAME categorical and numerical scores.

## Statistics

### Data collection and management

At the time of the NAME assessment, we collected information about the following variables: infants' gestational age (weeks), birth weight (grams), length at birth (cm), head circumference at birth (cm), Apgar at 1 min, Apgar at 5 min, weight at evaluation (grams), length at evaluation (cm), head circumference at evaluation (cm), mode of delivery (vaginal delivery or C-section), type of feeding (by suction, by gavage, or total parenteral nutrition), type of respiration (autonomous, non-invasive support, invasive support), need for oxygen.

We then assessed the presence of complications and, based on this collected data, we calculated a “complexity index,” i.e., an index that could easily discriminate between healthy and complicated infants. In particular, refining the procedure used in the paper where we assessed the validity of the NAME procedure (26), we constructed the complexity index as the sum of 10 subscores according to the rules described in Table 2.

**Table 2:** Complexity index calculation.

	**Score**		
**Domain**	**0**	**1**	**2**
Intrauterine growth disorder	Adequate for gestational age	Intrauterine growth restriction	Small for gestational age
Gestational age	Full-term (≥37 weeks)	Preterm (≥32 weeks)	Very late preterm (<32 weeks)
Birth weight	>2,500 gr	1,500–2,499 gr	<1,500 gr
Respiratory diseases	Autonomous respiration AND no need for oxygen	Respiration with non-invasive support (e.g., nCPAP/HFNC) OR with the need for oxygen	Respiration with invasive support (e.g., mechanical ventilation)
Cardiovascular diseases	No cardiovascular disease	Presence of cardiovascular disease	Presence of cardiovascular disease AND respiration with invasive support OR need for oxygen
Gastroenteric and uropoietic diseases	No gastroenteric disease AND no uropoietic disease	Gastroesophageal reflux OR necrotizing enterocolitis OR nephropathy	Gastroesophageal reflux OR necrotizing enterocolitis OR nephropathy AND need for surgery
Neurological diseases	No neurological disease	Gestational age <37 OR feeding by gavage OR total parenteral nutrition	Presence of neurological disease OR feeding by gavage OR total parenteral nutrition AND Gestational age >32
Infections	No infection	Sepsis	Septic shock OR meningoencephalitis
Metabolic diseases	No metabolic disease	Presence of metabolic alterations	Total parenteral nutrition
Genetic diseases or syndromes	No genetic disease or syndrome	Presence of life-compatible genetic disease	Presence of life-incompatible genetic disease (i.e., trisomy 13 or 18)

The complexity index is calculated by adding the scores obtained for each domain (e.g., if a newborn is preterm, they would have a score of 1 in the domain “Gestational age”). nCPAP: nasal continuous positive airway pressure; HFNC, High-flow oxygen through nasal cannula.

The 10 subscores regarded the following domains: (1) intrauterine growth disorders, (2) gestational age, (3) birth weight, (4) respiratory diseases, (5) cardiovascular diseases, (6) gastroenteric and uropoietic diseases, (7) neurological diseases, (8) infections, (9) metabolic diseases, and (10) genetic diseases or syndromes.

As every subscore can range from 0 (no complications) to 2 (severe complications), the complexity index ranges from 0 (no complications) to 20 (severe complications in every health domain). The complexity index was determined by the bedside neonatologist, who assessed the preterm based on the domains abovementioned. The evaluation took place before the NAME assessment. In fact, the complexity index was measured in the morning and the NAME score in the afternoon. This was assured to maintain blindness between operators.

Data were entered into a dedicated data warehouse that was created to record several types of information about the babies, including sex, gestational age, weight at birth, clinical conditions and side effects. The data operator was in charge of managing the data warehouse and collecting data into a dedicated in-house software, had full access to the system and did not take part in any clinical activities or manual procedure. The evaluators had access only to the assessment form, as well as the neonatologist in charge of sample selection was blinded about the NAME score having access only to the clinical neonatological data entry form.

Despite being a new index and, thus, in need of a proper validation, we chose to compute the complexity index because the literature lacks a general index that could be applied to any newborn, regardless of age or time of admission, and able to give a clear indication about the newborns' clinical conditions and complications. Although scores able to predict newborns' mortality and morbidity such as the Clinical Risk Index for Babies (CRIB) and Score for Neonatal Acute Physiology (SNAP) are available, these scores are usually applied within a few hours after admission to NICU (27, 28).

### Sample size calculation

Based on the data analyzed in the paper assessing NAME validity (26), we calculated the required sample size for the primary outcome, i.e., the correlation between the complexity index and the NAME categorical score. Therefore, using a correlation coefficient of −0.31, a confidence interval width of 0.19 and a level of significance of 0.05, the required sample size for the present study was *n* = 156. Indeed, a recent paper shows that, given the aforementioned parameters, a sample size of almost 200 subjects gives a power β of 80% (29).

### Statistical analysis

We described the general characteristics of the enrolled infants, using mean ± SD: gestational age, age at the time of NAME assessment, birth weight, length at birth, head circumference at birth, Apgar at 1 min, Apgar at 5 min, weight at evaluation, length at evaluation, head circumference at evaluation, mode of delivery, type of feeding, complexity index (subscores and total score). We then reported the number of female and male newborns and described the characteristics stratified by sex.

For assessing the primary outcome of the study, we calculated Kendall's τ correlation between the complexity index and the categorical NAME score. As in a previous paper, we also calculated the odds ratio (OR) between the complexity index and the categorical NAME score through an univariate unadjusted ordinal logistic regression.

We then performed several exploratory analyses to understand whether other variables could affect the NAME scores: in particular, we sought possible correlations between the collected data and both the categorical and numerical NAME performing Kendall's τ correlations for numerical data and Chi-squared tests for categorical data.

Data were analyzed using the free software R (Version 3.6.1, The R Foundation for Statistical Computing), specifically by loading the following packages: irr, lme4, presize, rel. Statistical significance was set for *p* ≤ 0.05.

## Results

### Sample characteristics

We enrolled 202 newborns, 92 (45.54%) female and 110 (54.46%) male, with a mean gestational age of 34.1 weeks (± 4.3) and a mean birth weight of 2,093.4 gr (± 879.8). At the time of the NAME assessment, the newborns were aged 35.9 weeks (± 4.1) and had a weight of 2,055.3 gr (± 750.6). Table 3 shows the other general characteristics of the sample population.

**Table 3:** General characteristics of the sample population.

	**Total**	**Female**	**Male**
*N* (%)	202 (100%)	92 (45.54%)	110 (54.46%)
Gestational age (wk)	34.1 ± 4.3	33.8 ± 4.7	33.3 ± 4.2
Age at evaluation (days)	12.7 ± 15.2	13.8 ± 18.1	11.7 ± 12.3
Birth weight (gr)	2,093.4 ± 879.8	1,990.8 ± 871.7	2,179.3 ± 881.4
Length at birth (cm)	43.4 ± 5.6	42.6 ± 5.8	44.1 ± 5.3
Head circumference at birth (cm)	30.5 ± 3.6	29.9 ± 3.9	31.0 ± 3.3
Apgar at 1 min	7.4 ± 2.0	7.1 ± 2.2	7.7 ± 1.9
Apgar at 5 min	8.9 ± 1.2	8.7 ± 1.3	9.0 ± 1.2
Weight at evaluation (gr)	2,533.0 ± 703.1	2,468.4 ± 687.4	2,587.0 ± 714.5
Length at evaluation (cm)	46.4 ± 3.6	46.3 ± 3.5	46.5 ± 3.8
Head circumference at evaluation (cm)	32.3 ± 1.8	32.3 ± 1.8	32.3 ± 1.8

Values shown are mean ± SD.

Table 4 shows the sample clinical characteristics, in particular, the type of delivery, the need for supported nutrition, and the pathologies suffered by the infants at the time of NAME assessment. Of the enrolled newborns, 69,80% had a vaginal delivery and 59.41% were on gavage. Metabolic, respiratory, and cardiovascular diseases, as well as intrauterine growth disorders, were the most representative pathologies (respectively, 57.43, 52.97, 27.23, and 35.15%). For a more detailed list of the infants' clinical conditions at the time of NAME assessment, see Supplementary Table 1.

**Table 4:** Clinical characteristics of the sample population at the time of NAME assessment.

**Characteristics**		**No. (%) of newborns**
		202 (100%)
Delivery	Vaginal delivery	141 (69.80%)
	C-section	61 (30.20%)
Feeding	By suction	58 (28.71%)
	By gavage	120 (59.41%)
	Total parenteral nutrition	24 (11.88%)
Intrauterine growth disorders	No	131 (65.85%)
	Yes	71 (35.15%)
Respiratory diseases	No	95 (47.03%)
	Yes	107 (52.97%)
Cardiovascular diseases	No	147 (72.77%)
	Yes	55 (27.23%)
Gastroenteric diseases	No	183 (90.59%)
	Yes	19 (9.41%)
Surgery	No	192 (95.54%)
	Yes	9 (4.46%)
Urogenital diseases	No	194 (96.04%)
	Yes	8 (3.96%)
Neurological diseases	No	192 (95.05%)
	Yes	10 (4.95%)
Metabolic diseases	No	86 (42.57%)
	Yes	116 (57.43%)
Genetic syndromes	No	197 (97.52%)
	Yes	5 (2.48%)
Infections	No	172 (85.15%)
	Yes	30 (14.85%)

The mean complexity index of the enrolled newborns was 5.9 (±2.8), with the subscore for neurological diseases being the highest one (1.3 ± 0.7), followed by the subscores for birth weight and gestational age (respectively, 1.0 ± 0.8 and 0.9 ± 0.8). On the contrary, the subscores for genetic syndromes (0.0 ± 0.2) and infections (0.1 ± 0.4) were the lowest ones (Table 5).

**Table 5:** Complexity index and its subscores.

	**Total**	**Female**	**Male**
*N* (%)	202 (100%)	92 (45.54%)	110 (54.46%)
Complexity index	5.9 ± 2.8	6.5 ± 2.8	5.9 ± 2.8
Gestational age	0.9 ± 0.8	1.0 ± 0.8	0.9 ± 0.8
Birth weight	1.0 ± 0.8	1.1 ± 0.8	0.9 ± 0.7
Intrauterine growth	0.5 ± 0.8	0.4 ± 0.8	0.5 ± 0.8
Respiratory diseases	0.7 ± 0.6	0.7 ± 0.6	0.7 ± 0.6
Cardiovascular diseases	0.5 ± 0.8	0.5 ± 0.8	0.4 ± 0.8
Gastro-urogenital diseases	0.2 ± 0.5	0.1 ± 0.4	0.2 ± 0.5
Neurological diseases	1.3 ± 0.7	1.3 ± 0.7	1.3 ± 0.8
Metabolic diseases	0.8 ± 0.7	0.7 ± 0.6	0.8 ± 0.7
Infections	0.1 ± 0.4	0.2 ± 0.4	0.1 ± 0.3
Genetic syndromes	0.0 ± 0.2	0.0 ± 0.1	0.0 ± 0.2

Values shown are mean ± SD.

### Correlations between NAME scores and sample clinical characteristics

Concerning the primary outcome, i.e., the correlation between the complexity index and the NAME categorical score, the Kendall's τ correlation was −0.206 [95% CI: (−0.292, −0.116), *p*-value < 0.001]. The corresponding OR was 0.838 [95% CI: (0.757, 0.924), *p*-value < 0.001], that is, the higher the complexity index, the worse the infant's conditions as revealed by the NAME assessment. This result held true for both categorical and numerical NAME (Table 6).

**Table 6:** Univariate statistical analyses between NAME scores and complexity index scores through Kendall tau correlation coefficient.

	**NAME categorical score**		**NAME numerical score**	
	**Kendall τ (95% CI)**	***P*-value**	**Kendall τ (95% CI)**	***P*-value**
Complexity index	−0.206 (−0.292, −0.116)	<0.001***	−0.193 (−0.280, −0.103)	<0.001***
Gestational age (score)	−0.201 (−0.288, −0.112)	<0.001***	−0.206 (−0.293, −0.117)	<0.001***
Weight at birth (score)	−0.206 (−0.293, −0.117)	<0.001***	−0.208 (−0.294, −0.118)	<0.001***
Intrauterine growth (score)	0.015 (−0.077, 0.106)	0.823	0.045 (−0.047, 0.136)	0.465
Respiratory diseases (score)	−0.179 (−0.266, −0.089)	0.006**	−0.190 (−0.277, −0.100)	0.002**
Cardiovascular diseases (score)	−0.041 (−0.132, 0.051)	0.533	−0.033 (−0.124, 0.059)	0.586
Gastro-urogenital diseases (score)	−0.094 (−0.185, −0.003)	0.154	−0.084 (−0.174, 0.008)	0.172
Neurological diseases (score)	−0.035 (−0.127, 0.057)	0.583	0.000 (−0.092, 0.092)	0.996
Metabolic diseases (score)	−0.083 (−0.173, 0.009)	0.201	−0.082 (−0.172, 0.010)	0.171
Infections (score)	−0.051 (−0.142, 0.041)	0.449	−0.053 (−0.144, 0.039)	0.394
Genetic syndromes (score)	−0.011 (−0.103, 0.08)	0.865	0.007 (−0.085, 0.099)	0.909

CI, confidence interval; NAME, neonatal assessment manual score. **p ≤ 0.01; ***p ≤ 0.001.

Among the complexity index subscores, the ones regarding gestational age and birth weight also showed negative correlations with both NAMEs with a *p* < 0.001 (it is important to remember that these subscores were constructed in a way that higher values mean the infants show more complex clinical conditions). The only other subscore with a statistically significant negative correlation (*p* < 0.01) with the numerical NAME was the respiratory diseases subscore (Table 6).

Regarding the exploratory analyses performed with the purpose of understanding whether other variables could affect the NAME scores, gestational age and birth weight correlated in a statistically significant way with both NAME scores: in particular, the higher the characteristics' respective values, the higher the NAME scores, i.e., the better the infants' general conditions. Instead, the age at the time of NAME evaluation did correlate with neither the NAME scores (Supplementary Tables 2, 3).

## Discussion

This research showed a statistically significant negative correlation between the complexity index and the NAME categorical score. Because the complexity index in the present study was calculated by evaluating both the number and severity of infant pathologies, such a finding may have an interesting clinical interpretation; that is, infants with severe clinical conditions are more likely to receive a score of “Marginal” or “Bad” than infants with less severe clinical conditions.

This result adds evidence to the NAME model's construct validity, but on a larger sample, it shows that the NAME score could be used to recognize an infant's clinical severity easily and quickly (90 s). Interestingly, even the NAME numerical score showed a negative correlation with the complexity index. This result opens a fascinating intrinsic nature of the NAME model as the categorical score should be used to communicate between NICU operators efficiently; indeed, the lexicon “Good-Marginal-Bad” is commonly used and understood, whereas the numerical score might have a more precise role in assessing the infants' conditions.

Indeed, future studies should evaluate whether NAME scores correlate with the infant's developmental trajectory or whether the NAME can predict the short-term evolution of the infant's clinical condition (e.g., minutes or hours). If data support these hypotheses, NAME will become a valuable tool for caring for infants, especially preterm ones.

Interestingly, we found negative correlations between the subscore of the complexity index regarding respiratory diseases and both the NAME scores. Breathing problems are particularly common in preterm newborns and clinical manifestations such as respiratory distress syndrome (RDS) can be a comorbidity of prematurity and affect the infant's survival (30). Since breathing is regulated by the brainstem and cortical areas related to both the ANS and the interoceptive network (e.g., the parabrachial nuclei and the nucleus of the solitary tract), and the diaphragm is a muscle whose activity can be easily felt through haptic perception (31–33), the severity of respiratory conditions could indeed affect the NAME scores.

These results advanced the clinical validity of the NAME, which was developed to quickly assess an infant's general condition, especially in NICUs, by evaluating how the infant's body responds to static touch (10). Static touch can elicit fibers that communicate with cerebral networks, including the interoceptive network (10), which orchestrates how the organism adapts to environmental stressors. This causes systemic changes in blood flow and muscle contraction or relaxation, which trained operators can feel (10). We expected, therefore, that the NAME score would correlate with the clinical conditions of the infants because prematurity affects the ANS and other vital systems (34–36).

Despite technological advances in NICUs and the development of machine learning-based monitors, (37), routine care is still manual (11). Several papers showed that the NICU can be a stressful environment (38, 39): therefore, widely used tools such as Test of Infant Motor Performance, Brazelton Neonatal Behavioral Assessment Scale, and Assessment of Preterm Infants' Behavior (23, 24, 40, 41), which need time to be completed, can worsen the stress suffered by infants with overloaded survival systems. Compared to these evaluations, the NAME is faster and can give information about the infant's actual conditions, potentially helping clinical decision-making, even in an emergency.

A limitation of the present study is that only newborns with gestational ages equal to or older than 29 weeks were included. Although the reason was to avoid possible negative effects of the NAME procedure on these infants' critical conditions, future studies should assess the NAME feasibility on more premature newborns.

Furthermore, it would also be central to understand how drugs (such as dexamethasone, paralytics, anesthetics, and anticholinergics, as well as maternal medications such as glucocorticoids or selective serotonin reuptake inhibitors) could affect the score obtained through the NAME procedure, since they can affect the functionality of the systems (for instance, the ANS) that we have hypothesized as central in the NAME procedure (10).

Another limitation regards the complexity index. We chose to compute this index as the available scores (e.g., CRIB or SNAP) able to predict newborns' mortality have to be used within a few hours after admission to NICU to be valid and reliable (27, 28). Nonetheless, future studies, including a Delphi study, should validate the complexity index by evaluating (1) whether such a score could be useful and (2) how it should be actually implemented.

Moreover, future studies should better evaluate even whether pathologies could affect the NAME score or, at least, understand to which extent they could affect it. For instance: a genetic disease could affect the NAME score since it could heavily impact the infant's clinical condition, but it is hardly conceivable that it could affect the evolution of the NAME score in a short or very short time since it does not change as fast as respiratory problems could.

Therefore, a potential objective for future research on the NAME could be to discriminate between conditions that could impact the NAME in the long-term (e.g., weeks), in the short-term (e.g., days or hours), in the very short-term (e.g., minutes or even seconds, in case of emergency), or not at all. Viceversa, future research should focus on understanding the meaning changes in the NAME scores have on the underlying clinical conditions of the infant.

In the paper evaluating the reliability of the NAME score, we found an interesting effect of sex on operators' concordance, namely that male infants were more easily recognized as “Good” than female infants (42). However, the present study found no correlation between the NAME scores and sex. Preterm males have a higher risk of infections and a higher mortality rate than preterm females (43–45), but sex may not affect the NAME score. Future research should clarify this issue.

## Conclusion

The NAME score helps NICU professionals assess infants' general health by evaluating their touch response. According to the study, the higher the NAME score, the better the infant's clinical conditions.

This result, obtained on a larger sample, extends previous findings of the NAME's validity; it also opens the path for future research evaluating whether the NAME can predict an infant's short-term clinical conditions.

NAME could improve NICU care if future evidence supports this hypothesis.

## Data availability statement

The raw data supporting the conclusions of this article will be made available by the authors, without undue reservation.

## Ethics statement

The studies involving human participants were reviewed and approved by Ethics Committee of the Vittore Buzzi Pediatric Hospital, Milan, Italy. Written informed consent to participate in this study was provided by the participants' legal guardian/next of kin.

## Author contributions

AM, MG, EL, SL, and PB: conceptualization, methodology, and writing—original draft preparation. FC and MC: formal analysis. AM, EL, SL, and PB: investigation. AM, MG, EL, SL, PB, FC, MC, and GL: writing—review and editing. GL: supervision. All authors approved the final manuscript as submitted and agree to be accountable for all aspects of the work.

## Conflict of interest

The authors declare that the research was conducted in the absence of any commercial or financial relationships that could be construed as a potential conflict of interest.

## Publisher's note

All claims expressed in this article are solely those of the authors and do not necessarily represent those of their affiliated organizations, or those of the publisher, the editors and the reviewers. Any product that may be evaluated in this article, or claim that may be made by its manufacturer, is not guaranteed or endorsed by the publisher.

## Abbreviations

ANS: autonomic nervous system
NAME: neonatal assessment manual score
NICU: neonatal intensive care unit
OR: odds ratio
RDS: respiratory distress syndrome.
