# Supplementary material for: Newborns' clinical conditions are correlated with the neonatal assessment manual scorE (NAME)
Source: Front Pediatr. 2022 Sep 9;10:967301. doi: 10.3389/fped.2022.967301 (PMC9500432; doi:10.3389/fped.2022.967301)
Supplement: Supplementary file 1 [file Data_Sheet_1.PDF]

Supplementary Tables

Table S1. List of infants' clinical conditions at the time of NAME assessment

Domain	Disease	No. (%) of newborns
		202 (100%)
Intrauterine growth disorders	Extremely low birth weight	20 (9.90%)
	Intrauterine growth restriction	21 (10.40%)
	Large for gestational age	5 (2.48%)
	Premature rupture of the membranes	2 (0.99%)
	Small for gestational age	43 (21.29%)
Respiratory diseases	Apnea	11 (5.45%)
	Bronchopulmonary dysplasia	4 (1.98%)
	Bronchial malformative obstruction	1 (0.50%)
	Congenital pulmonary airway malformation	1 (0.50%)
	Hemothorax	1 (0.50%)
	Interstitial emphysema	1 (0.50%)
	Pneumothorax	5 (2.48%)
	Pulmonary hypertension	6 (2.97%)
	Pulmonary/ventilatory insufficiency	6 (2.97%)
	Respiratory distress syndrome	87 (43.07%)
	Tachypnea	3 (1.49%)
Cardiovascular diseases	Aortic coarctation	2 (0.99%)
	Aortic ectasia	2 (0.99%)
	Arrhythmia	1 (0.50%)
	Atrial septal defect	7 (3.47%)
	Biventricular cardiac hypertrophy	2 (0.99%)
	Cardiopathy	2 (0.99%)
	Great artery transposition	1 (0.50%)
	Patent ductus arteriosus	27 (13.37%)
	Patent foramen ovale	14 (6.93%)
	Portal vein thrombosis	1 (0.50%)
	Right aortic arch	1 (0.50%)
	Ventricular septal defect	12 (5.94%)
Gastroenteric diseases	Feeding disturbances	4 (1.98%)

	Gastrectasis	1 (0.50%)
	Gastroesophageal reflux disease	7 (3.47%)
	Intestinal occlusion/coarctation	3 (1.49%)
	Jejunal stenosis	1 (0.50%)
	Necrotizing enterocolitis	3 (1.49%)
Surgery	Anorectal malformation	1 (0.50%)
	Biliary atresia	1 (0.50%)
	Diaphragmatic hernia	2 (0.99%)
	Duodenal atresia	1 (0.50%)
	Esophageal atresia	2 (0.99%)
	Omphalocele	1 (0.50%)
	Umbilical/inguinal hernia	2 (0.99%)
Urogenital diseases	Calyceal pyelectasis	1 (0.50%)
	Cryptorchidism	1 (0.50%)
	Hydrocele	1 (0.50%)
	Hypospadias	2 (0.99%)
	Renal dysplasia/agenesis	2 (0.99%)
	Urethral megameatus	1 (0.50%)
Neurological diseases	Brainstem immaturity	1 (0.50%)
	Cerebral hemorrhage	2 (0.99%)
	Leukomalacia	4 (1.98%)
	Ischemic hypoxia	3 (1.49%)
Metabolic diseases	Anemia	29 (14.36%)
	Cholestasis	2 (0.99%)
	Coagulopathy	1 (0.50%)
	Hematochezia	1 (0.50%)
	Hyperglycemia	3 (1.49%)
	Hypocalcemia	13 (6.44%)
	Hypoglycemia	35 (17.33%)
	Hypokalemia	2 (0.99%)
	Hyponatremia	3 (1.49%)
	Icterus	77 (38.12%)
	Neutropenia	2 (0.99%)
	Osteopenia	1 (0.50%)

	Polycythemia	3 (1.49%)
	Thrombocytopenia	3 (1.49%)
Genetic syndromes	Hirschsprung disease	1 (0.50%)
	George syndrome	1 (0.50%)
	Trisomy 21 (Down syndrome)	3 (1.49%)
Infections	Conjuntivitis	8 (3.96%)
	General infections	19 (9.41%)
	Sepsis	5 (2.48%)

Table S2. Univariate statistical analyses between NAME scores and categorical variables

	NAME categorical score		NAME numerical score	
	**Chi-squared**	**p-value**	**Chi-squared**	**p-value**
Sex	0.543	0.762	5.668	0.684
Mode of delivery	4.266	0.118	9.137	0.331
Type of feeding	8.464	0.076	13.830	0.611

Table S3. Univariate statistical analyses between NAME scores and numerical variables

	NAME categorical score		NAME numerical score	
	**Kendall τ (95% CI)**	**p-value**	**Kendall τ (95% CI)**	**p-value**
Gestational age	0.187 (0.097, 0.274)	<0.001***	0.187 (0.097, 0.274)	<0.001***
Age at evaluation	-0.066 (-0.156, -0.026)	0.243	-0.046 (-0.137, -0.046)	0.374
Birthweight	0.183 (0.093, 0.270)	<0.001***	0.178 (0.088, 0.266)	<0.001***
Length at birth	0.183 (0.093, 0.270)	0.001**	0.176 (0.086, 0.264)	<0.001***
Head circumference at birth	0.134 (0.043, 0.223)	0.016*	0.146 (0.055, 0.235)	0.005**
Apgar at 1 minute	0.031 (-0.061, 0.122)	0.601	0.057 (-0.035, 0.148)	0.295
Apgar at 5 minutes	-0.020 (-0.112, 0.072)	0.742	0.017 (-0.075, 0.108)	0.770
Weight at evaluation	0.046 (-0.046, 0.137)	0.404	0.037 (-0.055, 0.129)	0.465
Length at evaluation	0.063 (-0.029, 0.153)	0.369	0.024 (-0.068, 0.116)	0.703
Head circumference at evaluation	0.071 (-0.021, 0.162)	0.312	0.067 (-0.025, 0.158)	0.301

Legend: CI, confidence interval; NAME, neonatal assessment manual score.

* p ≤ 0.05

** p ≤ 0.01

*** p ≤ 0.001
